# Electrospinning of Nanofibers for Energy Applications

**DOI:** 10.3390/nano6070129

**Published:** 2016-07-02

**Authors:** Guiru Sun, Liqun Sun, Haiming Xie, Jia Liu

**Affiliations:** 1 Institute of Functional Materials, Faculty of Chemistry, Northeast Normal University, Changchun 130024, China; sungr955@nenu.edu.cn (G.S.); sunlq446@nenu.edu.cn (L.S.); 2 National & Local United Engineering Lab for Power Battery, Northeast Normal University, Changchun 130024, China

**Keywords:** electrospinning, nanofibers, lithium-based batteries, fuel cells, dye-sensitized solar cells, supercapacitors

## Abstract

With global concerns about the shortage of fossil fuels and environmental issues, the development of efficient and clean energy storage devices has been drastically accelerated. Nanofibers are used widely for energy storage devices due to their high surface areas and porosities. Electrospinning is a versatile and efficient fabrication method for nanofibers. In this review, we mainly focus on the application of electrospun nanofibers on energy storage, such as lithium batteries, fuel cells, dye-sensitized solar cells and supercapacitors. The structure and properties of nanofibers are also summarized systematically. The special morphology of nanofibers prepared by electrospinning is significant to the functional materials for energy storage.

## 1. Introduction

Energy conversion and storage devices have drawn significantly attention with fossil fuels depleting, climate change, and environment deterioration. Although some devices have realized commercialization, new breakthroughs are needed to improve their performance, including durability, harvest efficiency, power density and conversion efficiency.

Nanofiber (NF) materials have been extensively studied as constituent parts of energy conversion and storage devices. Currently, much effort has been devoted to developing nanostructured materials synthesized by different methods, including chemical vapor deposition [1], wet chemical synthesis [2], sol-gel templating [3], self-assembly [4], the molten salt method [5,6,7,8,9,10,11,12,13,14,15,16,17], the polymer precursor method [18,19,20,21,22,23] and electrospinning. Among these methods, electrospinning, as a versatile, efficient and low-cost method, has been used widely to synthesize many special NFs with different morphologies, including core-shell [24], hollow [25], multilayer [26], porous [27], etc. Combined with the high surface area, controllable porosity and ease of accessibility, the electrospun NFs have come to have important roles in various energy storage fields.

In this review, we focus specifically on the application of electrospun NF materials in lithium-based batteries, fuel cells, dye-sensitized solar cells and supercapacitors.

## 2. Electrospinning

The electrospinning technique is a versatile, efficient, and low-cost method for NFs’ fabrication, which traces its roots back to an initial patent on the fabrication of textile yarns by Formhals in 1934 [28]. However, electrospinning did not become a popular technique. This technique was not revived until the 1990s, when several research teams (especially that of Reneker) found that it could be used to prepare NFs from many organic polymer solutions [29]. After that, the electrospinning technique has been further developed.

**Figure 1:**
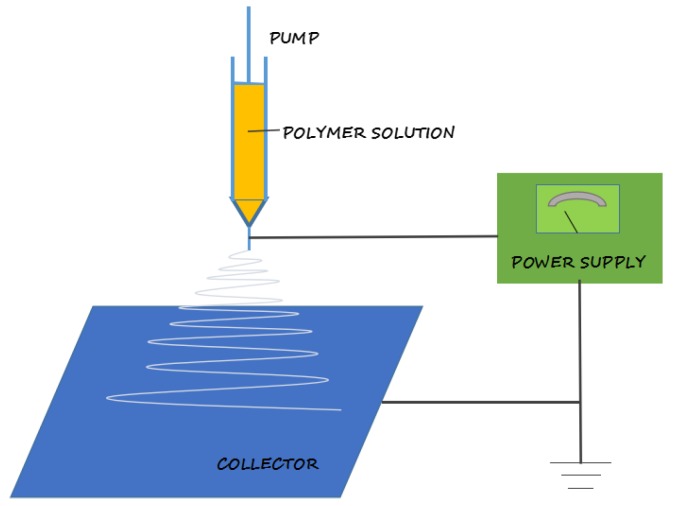
Schematic diagram of an electrospinning set-up.

A general electrospinning apparatus consists of a high voltage power supply, a syringe with a metallic needle with blunt-tip, and a grounded conductive collector, as shown in Figure 1. A high voltage electrostatic field is used to charge the surface of the polymer solution droplet. When the electrical forces overcome the surface tension and viscosity of the fluid, a liquid jet is ejected through the needle tip. The shape of the Taylor cone is influenced by the applied voltage. Within the evaporation of the mobile solution from the fluid jet to the collector, uniform NF mats form on the collector [30,31,32]. The types of collectors include a rotating drum, a metal frame, and a rotating mandrel, which can influence the alignment of electrospun NFs [33].

The properties of the fabricated NFs depend on the operating parameters, such as applied voltage, solution feed rate, spinning distance, environmental temperature, humidity, air velocity in the chamber, polymer molecular weight, solution conductivity, viscosity and surface tension. By adjusting the parameters, different morphologies of the NFs can be obtained [34,35,36]. The industrial development of electrospun NFs is at the starting stage. The NFs have potential in some fields, such as high temperature filtration, efficient catalysis, biology, tissue engineering, optoelectronic devices, and aerospace equipment [37].

## 3. Applications

Electrospun NF materials were applied widely in many fields due to their high surface area, good crystalline structure and superior kinetic property. In this section, we discuss the applications of electrospun NFs in lithium-based batteries, fuel cells, dye-sensitized solar cells and supercapacitors. The influences of the structures and the properties for NFs on these applications are also systematically summarized.

### 3.1. Lithium-Based Batteries

**Figure 2:**
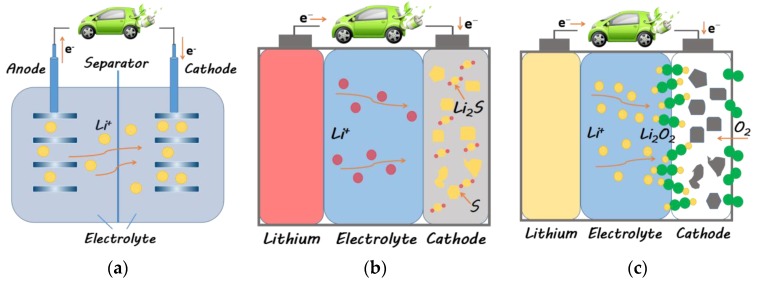
Schematics of: (**a**) Li-ion batteries; (**b**) lithium-sulfur batteries; (**c**) lithium-oxygen batteries.

According to the development course of lithium-based batteries, there are three critical battery systems, including Li-ion batteries (LIBs) (Figure 2a), lithium-sulfur batteries (LSBs) (Figure 2b) and lithium-oxygen (Li-O_2_) batteries (Figure 2c). As one of the most important energy storage systems, the Li-ion battery (LIB) has been used not only in portable electronics [38], but also in power batteries for electric vehicles now. Simultaneously, to meet the demands of all-electric vehicles in the long term [39], researchers have been devoted to developing other battery systems with lithium metal as the anode material to improve the energy density, such as LSB and Li-O_2_ batteries. Certainly, the application of NF materials on these three lithium-based batteries plays a fundamental role to improve the energy density. In this section, we focus on the application of NFs on the LIB, LSB, and Li-O_2_ battery systems.

#### 3.1.1. Li-Ion Batteries

At present, for the commercial LIBs, the electrode materials usually are based on powder materials. The long migration pathways for the Li^+^ of powder materials may lead to the large volume expansion occurring during cycling, resulting in the low rate of performance and poor cyclability [38]. NF materials are promising materials for LIBs because of their good electrochemical activity, high mechanical strength, and large specific surface area. In this section, recent advances in the areas of electrospun NFs as cathodes, anodes, and separator materials for LIBs are briefly summarized.

##### 3.1.1.1. Cathode Materials

LiCoO_2_ is the first commercial cathode material for LIBs [40]. Jiao et al. [41] synthesized LiCoO_2_ NFs via the sol-gel and electrospinning technique. The LiCoO_2_ NFs delivered first cycle discharge capacities of 182 mAh/g (powder-based electrodes with first cycle discharge capacities of 140 mAh/g). However, LiCoO_2_ NF electrodes exhibited a poor cyclability. In order to solve this problem, the core-shell LiCoO_2_-MgO NFs were prepared through a coaxial electrospinning [42]. The first cycle discharge capacity of core-shell LiCoO_2_-MgO NFs was close to that of the bare LiCoO_2_ NFs. After 40 cycles, the discharge capacity of the core-shell LiCoO_2_-MgO NFs electrode still remained at 90.0% of the initial value (bare LiCoO_2_ NFs with a capacity retention of 52.0%). LiCoO_2_-MgO NFs can effectively avoid the increasing of the impedance result from passive surface film formation during cycling.

As a ternary layered material, Li-Ni-Co-Mn-O has attracted considerable interest as the cathode material for LIBs to replace the commonly-used material LiCoO_2_ due to its high capacity, good thermal stability and excellent rate capability [43]. Currently, most prepared Li-Ni-Co-Mn-O have the Li^+^/Ni^2+^ cation mixing structure, resulting in depressing of the Li^+^ mobility, which leads to the poor cyclability of the cells [44]. In order to overcome this obstacle, electrospun LiNi_1/3_Co_1/3_Mn_1/3_O_2_ was fabricated, which was proven to be able to largely improve the cell performance [45,46,47,48]. Xiong et al. [45] prepared LiNi_0.5_Co_0.2_Mn_0.3_O_2_ with a particle size of 300 nm by the electrospinning method and LiNi_0.5_Co_0.2_Mn_0.3_O_2_ with a particle size of 1 um by the sol-gel method. The electrospinning material exhibited better performance than the sol-gel material, which is mainly attributed to the shorter Li^+^ and electron migration distance of the electrospinning material. LiNi_1/3_Co_1/3_Mn_1/3_O_2_ NFs were obtained by electrospinning poly(vinylpyrrolidone) (PVP) and MNO_3_ (M = Li, Ni, Co, Mn). The resulting LiNi_1/3_Co_1/3_Mn_1/3_O_2_ NFs showed a high first charge and discharge capacity of 217.93 mAh/g and 172.81 mAh/g, respectively [46]. Electrospun PVP and M(CH_3_COO)_2_ (M = Li, Ni, Co, Mn) fibers can yield a Li-rich Li_1.2_Mn_0.54_Ni_0.13_Co_0.13_O_2_-encapsulated carbon nanofiber (CNF) network (Li_1.2_Mn_0.54_Ni_0.13_Co_0.13_O_2_/CNF) that exhibited a high coulombic efficiency of 83.5% and a discharge capacity of 263.7 mAh/g [47]. The Li-rich Li_1.2_Ni_0.17_Co_0.17_Mn_0.5_O_2_ were also synthesized by the electrospinning technique, with PVP and polyvinyl acetate (PVAc) as different polymer sources [48]. The morphology of Li_1.2_Ni_0.17_Co_0.17_Mn_0.5_O_2_ NFs has depended on the different heat temperatures and the polymer used. The result shows that the porous nanostructure morphology can enhance the initial discharge capacity of the electrospun samples.

**Figure 3:**
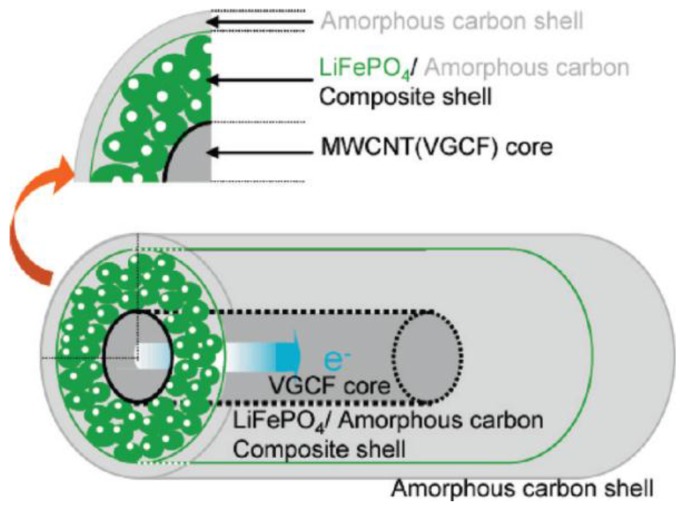
Model image of the triaxial nanowire with a vapor-grown carbon fiber (VGCF) core column and the two layer shells: an outer shell of amorphous carbon and an inner composite shell of LiFePO_4_ and amorphous carbon. Reproduced with permission from [53]. Copyright American Physical Society, 2010.

LiFePO_4_ was discovered by Goodenough in 1997, which possesses high discharge potential, large specific capacity (170 mAh/g) and good thermal stability [49]. However, LiFePO_4_ has low conductivity (~10^−9^ S/cm) and low rate capability due to the one-dimensional twisted channel of Li^+^ [50]. Particle nanocrystallization is supposed to be an effective method to shorten the diffusion channels. Thus, the LiFePO_4_/C composite NFs were prepared via electrospinning, with polyacrylonitrile (PAN) as polymer additives, which showed a good cyclability [51]. Interestingly, a slight increase of capacity was exhibited rather than the capacity fading after several cycles, which could be attributed to slow electrolyte penetration into the electrode or the crack formation on the amorphous carbon layer during cycling that increases the surface area of NFs and improves the electrode-electrolyte interaction. LiFePO_4_/carbon nanotube (CNT)/C composite NFs also were obtained via the combination of electrospinning and sol-gel methods, with PAN as the electrospinning media and carbon source [52]. The result shows that electrospinning is an effective method for minimizing the aggregation of LiFePO_4_ particles and promoting the formation of a conducting carbonaceous layer on the LiFePO_4_ surface. CNTs were well-dispersed in the carbonaceous matrix, which can help increase the electrochemical performance of the LiFePO_4_ cathodes by forming conducting bridges between LiFePO_4_ particles and enhancing the electron transport of the system. In another format, triaxial LiFePO_4_ nanowires (NWs) with a multiwall CNT core column and an outer shell of amorphous carbon were successfully synthesized with the electrospinning method [53]. The model image of the material is demonstrated in Figure 3. The CNT core oriented in the direction of the wire played an important role in the conduction of electrons during the cycling; the outer amorphous carbon shell suppressed the Fe^2+^ oxidation.

At the current stage, for a commercial battery, the cathode materials usually participate in a one-electron reaction, which inevitably limits the practical capacity [54]. Therefore, one promising approach to increase the delivered capacity is to develop a material that involves a two (or more)-electron reaction. Vanadium pentoxide (V_2_O_5_) has attracted particular attention because of the availability of the two-electron V^5+^–V^3+^ couple and its low cost, ease of synthesis, rich abundance, as well as its relatively high theoretical capacity (about 294 mAh/g with 2 Li^+^ insertions/extractions per unit formula) [55]. V_2_O_5_ NFs were synthesized by a hydrothermal treatment of electrospun PMMA and vanadium oxide NFs [56]. The delivered discharge capacity for the first cycle in an LIB with V_2_O_5_ NFs was 350 mAh/g. In addition, the capacity was maintained above 240 mAh/g after 25 cycles. The hierarchical V_2_O_5_ NFs with diameters of 200–400 nm were obtained via electrospinning combined with annealing, with PVP as the electrospinning media [57]. After 100 cycles, the specific capacity of the V_2_O_5_ NFs retains 133.9 mAh/g at a current density of 800 mA/g, corresponding to a high capacity retention of 96.05%. The ultra-long hierarchical V_2_O_5_ NWs with diameters of 100–200 nm were also prepared using PVA and low-cost raw materials by electrospinning combined with annealing [58]. The initial discharge capacities of the ultra-long hierarchical V_2_O_5_ NWs were up to 390 within a potential range of 1.75–4.0 V. In another study, electrospun PVP and a low-cost inorganic vanadium precursor can yield porous V_2_O_5_ NTs that showed good cycling performance; the capacity retention is 97.4% after 200 cycles at 50 C [59].

LiMn_2_O_4_ [60], Li_3_V_2_(PO_4_)_3_ [61,62] and LiNi_0.5_Mn_1.5_ [63,64,65] NFs as cathode materials were also prepared by electrospinning, which exhibited high capacity and good cyclability. This indicates that the NFs with a shorter Li^+^ and electron migration distance can provide fast Li^+^ intercalation and de-intercalation channels. The overview of some electrospun fibers as cathode materials of LIBs and their electrochemical performances is presented in Table 1.

##### 3.1.1.2. Anode Materials

Electrospun CNFs are popular anode materials for LIBs due to their low cost, easy availability and long cycle life [38]. However, LIBs with electrospun CNFs as the anode exhibited relatively low specific capacity and rate capability [66,67]. Therefore, flexible highly porous CNFs (HPCNFs) [68] and hollow CNFs (HCNFs) [69] were prepared by the electrospinning method. The HPCNFs delivered a reversible capacity as high as 1780 mAh/g after 40 cycles at current densities of 50 mA/g [68]. Controlling the mesopores in HCNFs is an effective means to improve the electrochemical performance of HCNFs [69]. HPCNFs and HCNFs with excellent electrochemical and mechanical properties result from the novel porous structure, which can provide the good access of the electrolyte to the electrode surface. Moreover, CNFs complemented with the high lithium storage capacity transition metals and transition metal oxides were used as anode materials for LIBs, which can result in a higher electrochemical performance; for example, Ag/CNFs composite by electrospinning PMMA/PAN and AgNO_3_ solution in DMF, where Ag NPs were embedded into the hollow CNFs [70]. The LIBs with Ag/CNFs composite as the anode delivered a capacity of 739 mAh/g at 50 mA/g, with 85% capacity retention after 100 cycles. Similarly, GeO_x_ NPs were encapsulated into hollow carbon shells using co-axial electrospinning [71]. This core-shell structure can alleviate the volume change of GeO_x_ during cycling, decrease the contact area between electrolyte and GeO_x_ to form a stable solid electrolyte interface film and increase the electrical conductivity. Cho et al. [72] prepared carbon-coated Fe_3_O_4_ hollow NFs (Fe_3_O_4_/C hNFs) and Fe_3_O_4_ hNFs via the electrospinning method, annealing and hydrothermal processing using PVP and Fe(NO_3_)_3_. The Li^+^ diffusion coefficient of the Fe_3_O_4_/C hNFs (8.0 × 10^−14^ cm^2^/s) is 60-times higher than that of Fe_3_O_4_ hNFs (1.33 × 10^−15^ cm^2^/s). The Fe_3_O_4_/C hNFs exhibit superior electrochemical performance compared to that of the Fe_3_O_4_ hNFs. CNF and transition metal oxide composites were also reported, such as Co_3_O_4_/CNF core-shell NWs [73], Mn_3_O_4_/CNF [74], GeO_2_/CNFs [75] and γ-Fe_3_O_4_/CNFs [76]. All of the composites are promising materials, which have important implications for developing high performance anodes for next-generation LIBs.

**Figure 4:**
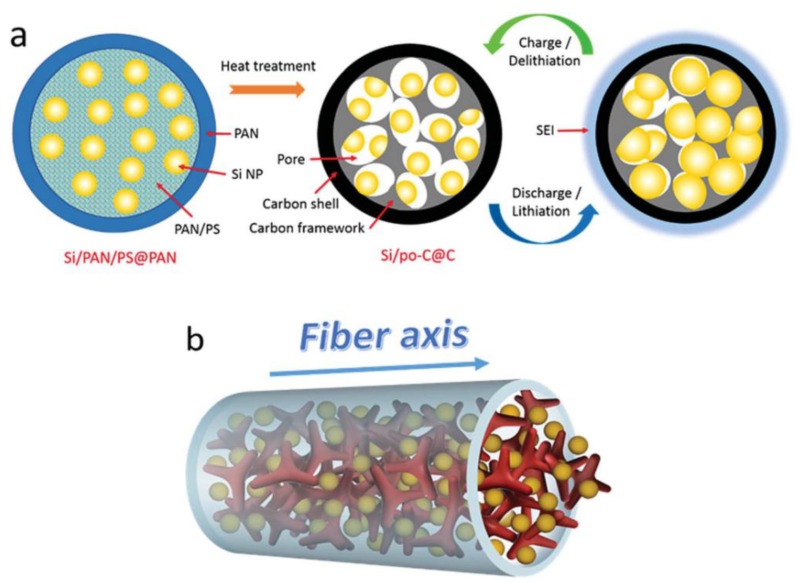
Schematics of the structural design of the core-shell silicon/carbon fiber (Si/po-C@C) composite fiber: (**a**) structural formation and change of Si/po-C@C during heat treatment and cycling process, respectively; (**b**) three-dimensional (3D) sketch of the overall structure. (SEI: solid-electrolyte interphase). Reproduced with permission from [82]. Copyright Royal Society of Chemistry, 2015.

**Figure 5:**
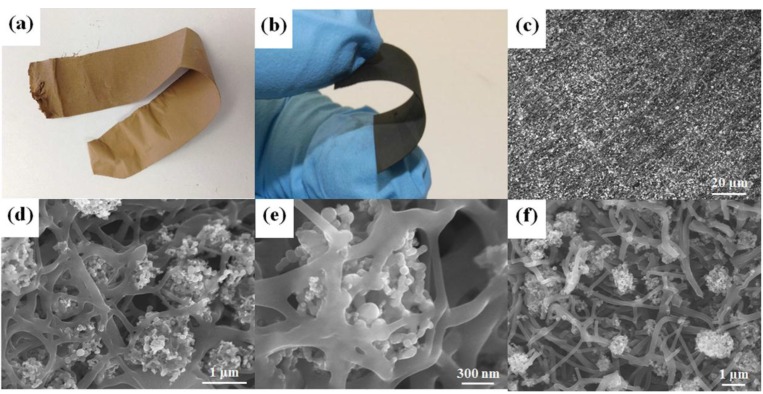
Photographs of the electrospun/sprayed flexible paper electrode (**a**) before and (**b**) after carbonization with 72 wt % Si; SEM images of the 3D Si/C fiber paper electrode: (**c**–**e**) top view and (**f**) cross-section. Reproduced with permission from [83]. Copyright Wiley, 2014.

Among anode materials for LIBs, silicon (Si) is the most promising candidate because of its high theoretical lithium storage capacity (~4200 mAh/g) [77]. Nevertheless, the pure Si anode, with the format of a nanoparticle (NP), NW or membrane, has faced adverse issues on electrode pulverization and capacity loss during frequent charging/discharging cycles [78]. Carbon/silicon composite was proved to exhibit a synergistic effect [79]. For example, Si/CNFs with mesopores were prepared by electrospinning PVA, aluminum acetylacetonate (AACA) and Si in DMF solution [80]. The mesoporous structure was generated by foaming NFs via AACA sublimation. The LIBs with Si/CNFs as the anode showed a superior high first discharge capacity of 1639 mAh/g and a good capacity retention of 83.6%. Xing et al. [81] synthesized pyrolytic carbon-coated Si/C NF (Si/C-CNF) composites by electrospinning and carbonization, which exhibited a high retention capacity of 1215.2 mAh/g after 50 cycles. An amorphous carbon layer covering the surface of Si/CNFs (prepared by electrospinning and sintering PAN and Si) can limit the volume expansion of the exposed Si NPs and prevent Si/CNF;s direct contact with the electrolyte. It also can create connections between the fibers that are conducive to Li^+^ ion transport. In other formats, a core-shell silicon/carbon fiber (Si/po-C@C) was prepared by simultaneously electrospinning polystyrene (PS)/PAN/Si as the core and electrospinning PAN as the shell then carbonization [82]. The structural design of the Si/po-C@C composite fiber is demonstrated in Figure 4. The LIBs with Si/po-C@C composite fiber as the anode exhibited a high initial discharge capacity of 997 mAh/g and a capacity retention of 71% after 150 cycles at a current density of 0.2 A/g. A novel flexible 3D Si/C fiber paper electrode was also fabricated via simultaneously electrospraying nano-Si/PAN clusters and electrospinning PAN fibers then carbonization [83]. The as-prepared Si/PAN paper and the carbonized Si/PAN paper (3D Si/C fiber paper) exhibited good flexibilities, as shown in Figure 5a,b, respectively. Figure 5c–f is the SEM micrographs of 3D Si/C fiber paper. When the novel structure of the 3D Si/C fiber paper was used as an anode for LIB, the LIB exhibited a high capacity (1600 mAh/g), an excellent rate capability performance and a low capacity loss (0.079% after 600 cycles).

**Scheme 1:**
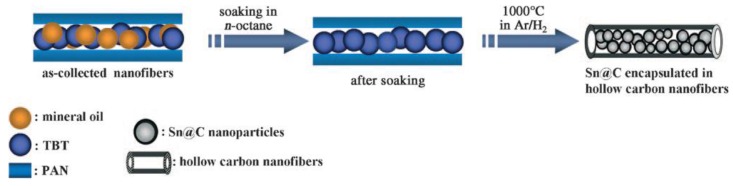
Preparation of Sn@carbon nanoparticles encapsulated in hollow carbon nanofibers. Reproduced with permission from [88]. Copyright Wiley, 2009.

As an anode material for LIBs, tin (Sn) has attracted considerable interest due to its high theoretical capacity (990 mAh/g) [84]. However, the volume changes during cycling and the tin NPs aggregate during the discharging process, resulting in the Sn-based materials’ poor cyclability [85]. To address these problems, Sn NP-loaded CNF (Sn NP/CNF) [86] and Sn NP-loaded PCNF (Sn/PCNF) [87] composites were fabricated. The discharge capacity for LIB with Sn/PCNF composite was 774 mAh/g at a high current density of 0.8 A/g after 200 cycles [87]. Wang et al. [88] prepared Sn@carbon NPs encapsulated in bamboo-like hollow CNFs (see Scheme 1) by pyrolysis of coaxial tributyltin (TBT)/PAN electrospun NFs, which contained Sn and C with a weight ratio of 7:3. The LIBs with the composite material as the anode displayed a high discharge capacity of 737 mAh/g after 200 cycles at 0.5 C. The high cyclability and capacity retention ratio could be attributed to providing the appropriate void volume of the composite to respond to the large volume change and to prevent pulverization of the Sn NPs. The 3D Sn/C hybrid core/shell NFs with micro-/nano-channels were also prepared by electrospinning, annealing and glucose-hydrothermal processing, with PAN as the electrospinning media and carbon source [85]. The LIBs with the obtained Sn/C hybrid NFs as the anode displayed a high initial discharge capacity of 1090.8 mAh/g at a current density of 40 mA/g and a high coulombic efficiency of nearly 100%. The advantages of this particular 3D core/shell architecture result from the greatly enhanced lithium storage from enlarging the electrode-electrolyte contact area, shortening the Li^+^ migration pathways and enhancing the Li^+^ and electrolyte diffusion of the active materials during cycling processes.

**Scheme 2:**
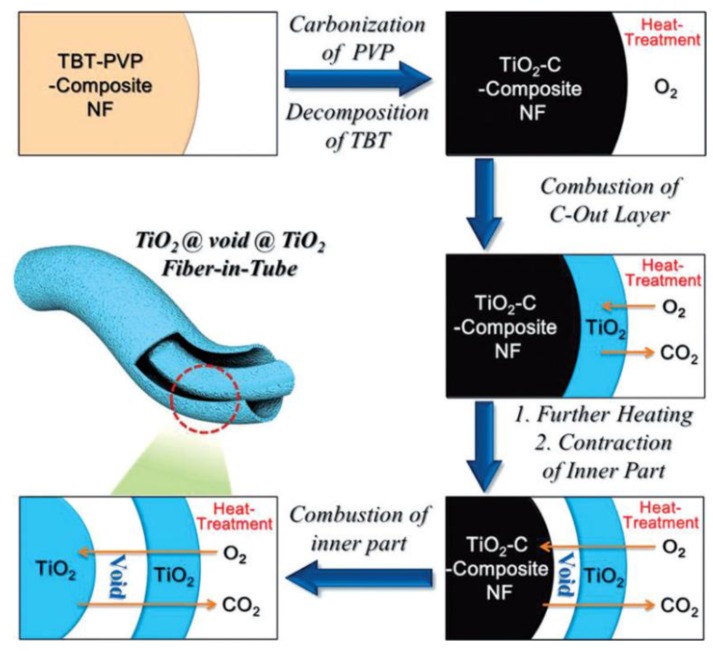
Formation mechanism of the TiO_2_ nanofiber with a fiber-in-tube nanostructure from the tributyltin (TBT)-poly(vinylpyrrolidone) (PVP) composite nanofiber. Reproduced with permission from [92]. Copyright Wiley, 2015.

As a promising candidate to replace graphite, metal oxide is believed to contribute to increasing energy density and decreasing cost [89]. TiO_2_ is one of the most exciting anode candidates for LIBs due to its high abundance, nontoxicity and structural integrity during cycling [90]. Nevertheless, TiO_2_ possesses low intrinsic electronic conductivity, which limits its application [91]. In order to improve the shortcoming, the TiO_2_ NFs were synthesized by electrospinning [91,92,93,94,95]. Sigmund et al. [91] obtained the electrospun TiO_2_ NFs with anatase and anatase-rutile mixed phase crystallites without carbon coating and then calcined the electrospun TiO_2_ NFs under different atmospheres. The results show that argon-calcined TiO_2_ NFs exhibited several orders of magnitude higher electronic conductivity. The anatase TiO_2_ NF with a fiber-in-tube structure was also reported, with the formation mechanism as shown in Scheme 2, which exhibited high structural stability during cycling [92]. Ramakrishna et al. [96] studied electrospun TiO_2_ nanostructures and their composites with multiwalled CNTs for rechargeable LIBs. The TiO_2_/CNT composite showed enhancement in the capacity retention (10–800 cycles) by increasing the retention from 81% to 92%. To improve the rate capability of TiO_2_ NFs, the metal oxide/TiO_2_ was prepared via electrospinning, such as Co_3_O_4_/TiO_2_ [93], which exhibited high discharge capacity and an excellent rate capability, resulting from the synergetic effect between Co_3_O_4_ and TiO_2_, as well as the unique feature of hierarchical heterostructures.

The Nb_2_O_5_ was also used as the anode material for LIBs [97]. The polymorphs of Nb_2_O_5_, including pseudohexagonal (H-Nb_2_O_5_), orthorhombic (O-Nb_2_O_5_) and monoclinic (M-Nb_2_O_5_), were synthesized by electrospinning [98]. After annealing, the H-Nb_2_O_5_ and O-Nb_2_O_5_ phases maintained the fibrous morphology, whereas the M-Nb_2_O_5_ phase adopted a distorted nugget structure. The M-Nb_2_O_5_ delivered the highest capacity and exhibited the highest capacity retention compared to the other two phases. The Li^+^ diffusion coefficients of H-Nb_2_O_5_, O-Nb_2_O_5_ and M-Nb_2_O_5_ were also studied, which were in the ranges of 10^−17^–10^−16^, 10^−15^–10^−14^ and 10^−13^–10^−12^ cm^2^/s, respectively [99]. The overview of some electrospun fibers as anode materials of LIBs and their electrochemical performances is presented in Table 2.

##### 3.1.1.3. Separator Materials

The traditional commercial separators for LIBs are microporous membranes, whose low porosity, unsatisfactory thermal stability and poor wettability in liquid electrolytes inevitably limit the battery performance [100]. In this regard, fiber-based membranes and fiber-coated membranes have been developed [101]. Electrospun poly(vinylidene fluoride) (PVdF)-based membranes as separators for LIBs have been reported and show advantages in electrochemical performances [102,103]. Owing to the softening of PVdF fibers at high temperatures, the electrospun PVdF-based membranes can form an interconnected web structure by annealing. The interconnected web structure greatly improves the physical properties and electrochemical performance [102]. Porous PVdF fiber-based membranes possess good electrochemical properties, such as a high ionic conductivity of 1.0 × 10^−3^ S/cm and a wide electrochemical stability window up to 4.5 V vs. Li^+^/Li at room temperature [103]. Furthermore, the electrospun poly(vinylidene fluoride-co-hexafluoropropylene) (PVdF-HFP) membranes possessed high electrolyte uptake and ionic conductivities (10^−3^ S/cm) [104]. However, PVdF and PVdF-HFP separators also have their disadvantages, such as the inferior thermal stability and intrinsically hydrophobic character. Wei et al. [105] prepared cellulose/PVdF-HFP membranes by coaxial electrospinning with superior thermal stability and a hydrophilic property. Other electrospun PVdF-based materials, such as P(VdF-HFP)/SiO_2_ [106,107], PVdF-HFP/PMMA [108], P(VdF-co-HFP)/PAN [109] and PVdF/SiO_2_-Poly(acrylic acid) lithium (PAALi) [110] as separators for LIBs, all delivered high ionic conductivity, good tensile strength and high uptake/leakage. These results show that the physical properties and electrochemical performance of the membranes can be improved by adding SiO_2_, PMMA and PAN [106,107,108,109,110]. Electrospun PAN-based NF membranes are another common host separator for LIBs due to their high ionic conductivity, electrolyte uptake and electrochemical stability window [111,112]. Sui et al. [113] prepared PMMA/PAN NF membranes with a core-shell structure by electrospinning. The LIBs with the PMMA/PAN NF membranes as the separator exhibited excellent initial discharge capacities, as well as remarkable cycle performance, resulting from the unique electrospun core-shell structure of PMMA/PAN NF membranes. Electrospun SiO_2_/PAN [114,115], PAN/Al_2_O_3_-TEGDA-BA [116] (triethylene glycol diacetate-2-propenoic acid butyl ester (TEGDA-BA)), PAN/TEGDA-BA [117], PAN/PEO [118] (polyethylene oxide (PEO)), oxy-PAN [119] and LLTO/PAN (lithium lanthanum titanate oxide (LLTO)) [120] NF membranes were used as separators for LIBs, which showed remarkably good physical properties and electrochemical performance. The overview of electrospun fiber membranes as separators of LIBs mentioned above is presented in Table 3.

#### 3.1.2. Li-S and Li-O_2_ Batteries

##### 3.1.2.1. Li-S Batteries

Sulfur possesses the characteristics of high natural abundance, low cost and environmental friendliness. Equally, as a promising electrode material for lithium secondary batteries, sulfur has attracted attention because of its high theoretical specific capacity (1675 mAh/g) and high theoretical specific energy (2600 Wh/kg) [122]. Sulfur, working as the cathode of Li-S batteries, has an insulating property [123]. In recent years, though some progress has been made in LSBs, the dissolution of intermediates (Li_2_S*_x_*, 3 ≤ *x* ≤ 8) and the rapid capacity decay limit the future application of LSBs [124,125]. In order to overcome the above disadvantages, various kinds of carbon materials were used to composite with sulfur. Electrospinning is an effective method to obtain carbon materials with a special morphology for the storage of sulfur. The porous carbon NF (PCNF)/S nanocomposites were fabricated by carbonization of electrospun PAN/PMMA NFs and the subsequent chemical reaction-deposition strategy [126]. The as-prepared nanocomposites have a high electrical conductivity and extremely high surface area. The LSBs with the PCNF/S nanocomposites as cathodes showed high reversible capacity, good discharge capacity retention and an excellent rate capability because the sulfur was dispersed and immobilized on the porous structures of CNFs, alleviating the polysulfide shuttle phenomenon. A hierarchical structure carbon/sulfur composite is fabricated based on electrospun CNF matrices [123]. The CNF matrices were obtained by removing Ni NPs of Ni/CNF, which was synthesized by electrospinning PAN and Ni(AC)_2_ H_2_O. The cell with the carbon/sulfur composite as the cathode exhibited an initial discharge capacity of 845 mAh/g at 0.25 C, with a retention of 77% after 100 cycles. Guo et al. [127] prepared the ordered mesoporous carbon fiber/sulfur (OMCF)/S composite via electrospinning. The OMCF/S composite with a weight ratio of 3:7 delivered a stable discharge capacity of 690 mAh/g after 300 cycles at 0.3 C. In situ synthesis is the most preferred preparation method of cathode materials for LSBs, particularly in synthesizing precursor mixture materials by electrospinning [128]. PAN NFs were fabricated in situ firstly with a porous structure by electrospinning, and then, the final product, PCNFs, was obtained by the following carbonization [129]. The prepared PCNFs were both mesoporous and microporous with large surface areas, which can store much sulfur and were utilized as conductive matrices for sulfur to form PCNFs/S nanocomposites. The LSB with the PCNFs/S nanocomposites as the cathode showed an initial discharge capacity of 1155 mAh/g at 0.02 C.

##### 3.1.2.2. Lithium-O_2_ Batteries

Recently, a rechargeable Li-O_2_ battery has attracted significantly attention due to its high theoretical energy density (11,680 Wh/kg) [130]. The Li-O_2_ battery involves a distinct operating mechanism (typically, 2Li^+^ + O_2_ + 2e^−^ ↔ Li_2_O_2_, E^0^ = 2.96 V vs. Li/Li^+^) on the O_2_ electrode, which relates to the Li_2_O_2_ formation during the discharging process (oxygen reduction reaction = ORR) and the Li_2_O_2_ decomposition during the charging process (oxygen evolution reaction = OER) [131,132]. To date, the O_2_ electrode is porous, which can provide the solid-liquid-gas tri-phase and electron migration regions for the ORR and OER. Therefore, the performances of the Li-O_2_ battery depend on the material and architecture of the O_2_ electrode [133]. Electrospinning is an attractive method for fabricating the materials of the O_2_ electrode for Li-O_2_ batteries [134].

In recent years, numerous electrospun electrocatalyst have been explored to enhance the performance of Li-O_2_ batteries. For example, Kim et al. [135] prepared the 1D Co_3_O_4_ NFs (obtained by electrospinning and calcination of PVP and Co precursor) immobilized on both sides of 2D non-oxidized graphene nanoflakes as a bifunctional composite catalyst for an O_2_ electrode in Li-O_2_ batteries. It showed the first discharge capacity of 10,500 mAh/g and a stable cyclability of 80 cycles with a limited capacity of 1000 mAh/g. 1D porous La_0.5_Sr_0.5_CoO_2.91_ NTs were synthesized via electrospinning and sintering PVP and La(NO_3_)_3_·6H_2_O, Sr(NO_3_)_2_ and Co(CH_3_COO)_2_·4H_2_O [134]. The resulting La_0.5_Sr_0.5_CoO_2.91_ NTs as O_2_ electrode catalyst exhibited a high initial discharge capacity of 7205 mAh/g at a current density of 100 mA/g and a stable cyclability of 85 cycles with a limited discharge depth of 1000 mAh/g. A hierarchical mesoporous/macroporous La_0.5_Sr_0.5_CoO_3 − *x*_ NTs (HPN-LSC) was also prepared by electrospinning PVP and La(NO_3_)·6H_2_O, Sr(NO_3_)_2_ and Co(NO_3_)_2_·6H_2_O [136]. The Li-O_2_ battery with HPN-LSC as the O_2_ electrode catalyst enhanced the reversibility and round-trip efficiency.

**Scheme 3:**
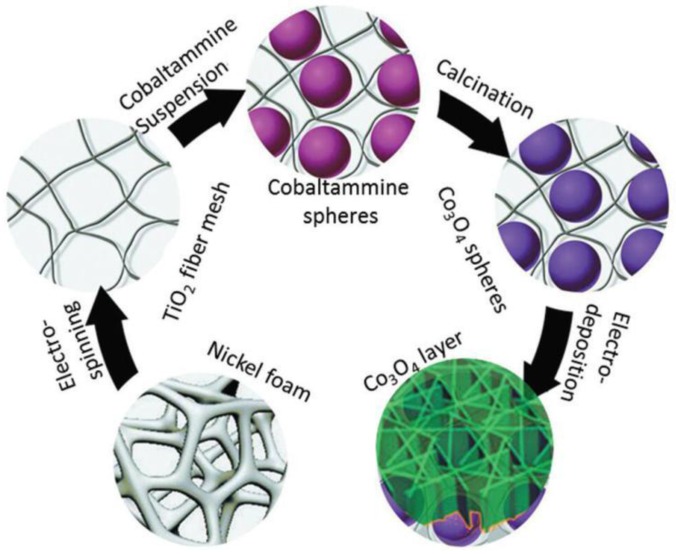
Schematic illustration of the typical preparation procedure for the Co_3_O_4_-based binder-free (CCTN) electrode. Reproduced with permission from [138]. Copyright Royal Society of Chemistry, 2015.

The O_2_ electrode architecture also influences the performance of Li-O_2_ batteries. To avoid the negative influence of the binder on the long-term stability, a novel “binder-free” design has been proposed [137]. Typically, electrospun PAN and Co(NO_3_)_2_·6H_2_O can yield the Co_3_O_4_/CNF composites, that as the O_2_ electrode exhibited a high initial discharge capacity of 760 mAh/g [137]. In another study, a Co_3_O_4_-based binder-free O_2_ electrode was reported for Li-O_2_ batteries [138]. Scheme 3 illustrates a typical procedure for the fabrication of the Co_3_O_4_-based binder-free O_2_ electrode. The Co_3_O_4_ nanoparticles formed nanoflakes by the aggregation in O_2_ electrode, which can provide a large amount of catalytic active sites for ORR/OER. The Co_3_O_4_ spheres embedded in the TiO_2_ fiber mesh may function as anchors to prevent the detachment of the Co_3_O_4_ layer from the current collector, resulting in excellent structural and cycling stability.

### 3.2. Fuel Cells

Fuel cells are energy conversion devices that can convert chemical energy into electrical energy through electrochemical oxidation of fuel (hydrogen or hydrogen-rich fuel). Fuel cells include proton exchange membrane fuel cells (PEMFCs) [139], direct methanol fuel cells (DMFCs) [140], alkaline fuel cells (AFCs) [141], phosphoric acid fuel cells (PAFCs) [142], solid oxide fuel cells (SOFCs) [143] and molten carbonate fuel cells (MCFCs) [144].

#### 3.2.1. Electrode Materials

The catalyst is the key material in fuel cells. It has been proven that the employment of a catalyst can largely influence the fuel cell performance [145]. Currently, electrospun NF materials have been used as catalyst and catalyst supporting materials for fuel cells because of their high poisoning resistance, high activity and good durability [146]. The PVP/Pt composite fibers were synthesized via electrospinning. After calcining, the Pt NWs with a high surface area were obtained, which displayed large electrochemically-active surface areas (ESA) of 6.2 m^2^/g and a high specific activity toward a methanol oxidation reaction (MOR) of 1.41 mA/cm^2^ in DMFCs [140]. Pt/Rh NWs [145] and Pt/Ru NWs [147] were further synthesized, which exhibited higher catalytic activities in fuel cells than that of the traditional NP catalyst. It has been also reported that the bimetallic NWs electrocatalysts showed better stability than the bimetallic NPs [147]. Nanoporous Pt/Fe alloy NWs [148] and Pt/Co alloy NWs [149] were synthesized and studied. The Pt/Fe alloy NWs’ overall wire diameters are about 10–20 nm, which showed a high specific activity (2.3-times that of conventional Pt/C catalyst) [148]. Pt/Co alloy NWs’ average diameters are 28 nm, which showed excellent stability activity to the ORR in acidic electrolytes [149].

It has been proven that the catalytic activity of the catalyst depends not only on the shape, size and distribution of the catalyst materials, but also on the categories and properties of the catalyst support [150]. The catalyst particles are dispersed on various supports, including CNFs, SnO_2_ and TiO_2_. Among them, CNFs are the most widely used catalyst supports. For example, Pt/CNF mats were prepared by electrospinning [146]. The catalytic peak current on the optimum Pt/CNF mat electrode reaches 420 mA/mg (the catalytic peak current of methanol oxidation on a commercial catalyst of 185 mA/mg). The results suggest that the electrospun CNF mats are favorable to enhance the performance of the catalyst. However, the cost and scarcity of Pt limit its application in fuel cells. Therefore, the low-cost N-doped CNFs were synthesized to replace Pt/CNFs [151,152,153]. Yu et al. [151] investigated the effect of different nitrogen sources (including melamine, aniline, urea and polyaniline) and nitrogen content on the activity of electrocatalyst, indicating that the best electrocatalytic activity was obtained with an aniline/PAN with a mass ratio of 20:3. You et al. [152] and Huang et al. [153] used NH_3_ as the nitrogen source to prepare the N-doped CNFs by carbonizing electrospun polyacrylonitrile (PAN) NFs in NH_3_. Metal-/alloy-doped CNFs were also used as catalysts for fuel cells, such as Ag [154], Co/N [155,156], Cu/Co [150], Fe/Co [157,158], Ni/Cu alloy [159], Fe/N [160,161] and Ni/Co [162] -doped CNFs. For example, Smirnova et al. [154] fabricated Ag/CNFs with different Ag loading amounts of 11, 15 and 25 wt %. The well-dispersed Ag NPs were attached to the surface of the electrospun CNFs. The results show that the mass activity of the 15 wt % Ag/CNFs system was the highest (119 mA/mg).

**Figure 6:**
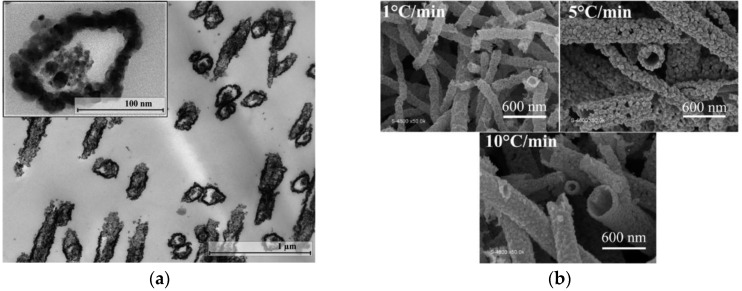
(**a**) TEM micrographs of microtomed sections of 5 at. % Nb-SnO_2_ loose tubes obtained using 8.5 *w*/*v* % SnCl_2_. Inset: close-up of the cross-section of a loose-tube fiber. (**b**) Loose tubes of niobium-doped SnO_2_ (8.5 *w*/*v* % SnCl_2_ and 5 at. % Nb) prepared using heating rates of 1, 5 and 10 °C/min during calcination. Reproduced with permission from [165]. Copyright American Chemical Society, 2013.

The transition metal oxides possess high chemical stability, good corrosion resistance and strong metal support interaction, like SnO_2_ and TiO_2_, which can enhance the activity of the catalysts. As catalyst supports, they are able to stabilize and disperse adequately a number of active phases [163]. Electrospun Pt/SnO_2_ NFs were used as the anode electrocatalyst material for PEMFCs [164], which exhibited a high electrochemically-active surface area (81.17 m^2^/g-Pt). A diffusion-limited current was achieved at 0.07 V. Cavaliere et al. [165] prepared the Pt supported on the electrospun Nb-SnO_2_ with a loose-tube (fiber-in-tube) morphology (Figure 6), which exhibited a higher electrochemical stability than conventional Pt/C electrodes. Cavaliere et al. [166] also synthesized Pt supported on electrospun Nb-SnO_2_ with a loose-tube structure as the cathode material for fuel cells, who studied in situ fuel cell operation under accelerated stress tests and confirmed that the voltage loss was negligible degradation. Electrospun Pt/TiO_2_ NFs were also synthesized by electrospinning and the reductive impregnation method, which were used as electrocatalysts and exhibited high activities for methanol oxidation [167]. Electrospun Nb-TiO_2_ NFs were investigated as electrocatalyst supports for PEMFCs [168]. The Pt/Nb-TiO_2_ NFs showed a high stability after accelerated stress tests, retaining 73% of the electroactive Pt area (the value of Pt/CNFs was 8%).

The membrane electrode assembly (MEA) is one of the important parts for fuel cells, which consists of the membrane and electrocatalyst [169]. Electrospinning is a straightforward method to prepare NFs of MEA for fuel cells. Electrospun nafion/polyphenylsulfone (PPSU) mats were synthesized for the H_2_/Br_2_ fuel cell [169], which exhibited good selectivity and high ionic conductivity. Shul et al. [170] fabricated electrospun SiC webs as membranes. The functionalized SiC webs (by introducing the hydroxyl group and phosphoric acid) showed a 70% better ion-exchange capability than that of the conventional membrane. The Pt NWs deposited on electrospun carbon film with a hierarchical structure were also used as MEA for fuel cells [171], which can induce the catalytic reaction toward formic acid electrooxidation.

#### 3.2.2. Electrolyte Membrane

The proton exchange membrane (PEM) is an electrolyte that behaves as an important indicator of the performance of fuel cells [172]. Nafion has been regarded as the most commonly-used polymer electrolyte membrane, which showed excellent chemical and physical stabilities and high proton conductivity [173]. However, the pure nafion is not a good candidate for electrospinning due to its low viscosity [174]. Aiming at the enhancement of the mechanical strength of the composite membrane, matrix polymers, such as PEO/Nafion [175] and PVA/Nafion [174], have been studied. To improve the proton conductivity, Na et al. [176] fabricated the sulfonated poly(ether ether ketone ketone) (SPEEKK) membrane with a spherical structure by electrospinning. The sulfonated SPEEKK membrane exhibited a high proton conductivity (0.37 S/cm), which is 37-times higher than that of the common SPEEKK membrane. The Nafion and PVdF composite membranes were fabricated by electrospinning [177]. They studied the Nafion NFs embedded in a PVdF matrix (N(fibers)/PVdF) and PVdF NFs embedded in a Nafion matrix (N/PVdF(fibers)). Both membrane structures exhibited similar in-plane conductivity, where the conductivity scaled linearly with the Nafion volume fraction. Hybrid organic-inorganic membranes were also prepared by electrospinning, which exhibited good thermal stability and a high proton conductivity of 15 ms/cm at 120 °C [178]. Shanmugam et al. [179] fabricated TiO_2_, CeO_2_ and ZrO_1.95_ NTs with mesoporous structures as fillers in the composite membranes, which were then incorporated in a Nafion ionomer to increase the water retention capability in the Nafion membrane.

### 3.3. Dye-Sensitized Solar Cells

Dye-sensitized solar cells (DSSCs) as the third generation of solar cells have attracted much attention due to their high energy efficiency, clean energy source and low cost, which can be applied to many portable and mobile ubiquitous power sources [180]. DSSCs consists of a photoanode, counter electrodes, dye and electrolyte [181]. NFs have been considered to be the most important material for DSSC photoanodes, counter electrodes and electrolytes.

#### 3.3.1. Photoanode

**Figure 7:**
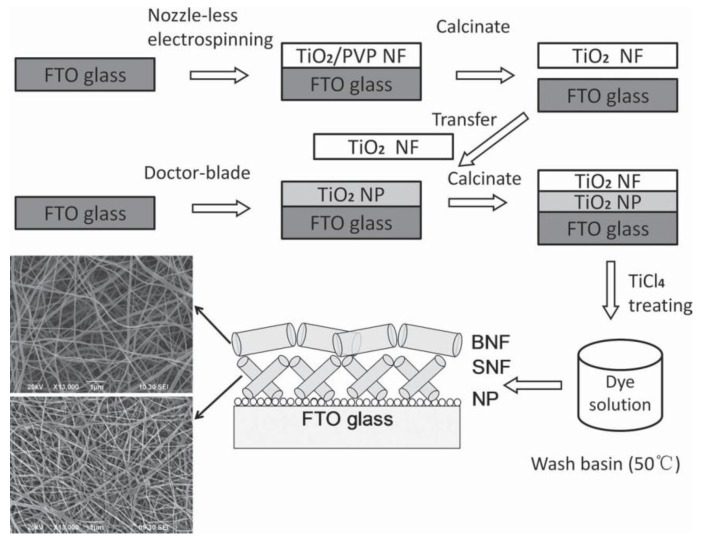
Schematic diagram of the fabrication procedure and SEM images of the bilayer TiO_2_ nanofiber photoanode (BNF: bigger-diameter nanofiber; SNF: smaller-diameter nanofiber). Reproduced with permission from [187]. Copyright Wiley, 2011.

In the DSSCs, the photoanode is a transparent conducting oxide coated with a film of a high band gap metal oxide (TiO_2_, ZnO, SnO_2_ and Nb_2_O_5_) [182,183]. Electrospun metal oxide NFs as the film coated with photoanode have been studied due to their high specific area [184,185,186]. Leung et al. [187] fabricated an innovative bilayer TiO_2_ NF photoanode by electrospinning. The bilayer TiO_2_ NF photoanode can offer excellent dye-loading, light harvesting and electron-transport properties, which improved the power conversion efficiency. Figure 7 shows the schematic diagram of the fabrication procedure for the bilayer TiO_2_ NF photoanode. In general, the photoanode with high specific surface area can improve light-scattering effects and facilitate electrolyte diffusion [188]. In this regards, Jang et al. [189] synthesized the multiscale porous TiO_2_ NFs via electrospinning, which were used as photoanodes of DSSCs. In another study, CNT/TiO_2_ NF [190] was fabricated by electrospinning. When 5 wt % of hollow CNTs was added into TiO_2_ NFs, the energy conversion efficiency of DSSCs reached 3.39%. Ko et al. [191] prepared a novel nanostructured PVdF NFs/TiO_2_ NP composite by electrospinning. The composite showed an outstanding bending stability because the PVdF NFs can reinforce the photoelectrode. Electrospun hollow TiO_2_ NFs were also obtained to enhance the conversion efficiency of ZnO-based DSSCs [192]. The results show that the weight of TiO_2_ influences the performance of the TiO_2_/ZnO composite photoanode. The maximal energy conversion efficiency reached 4.59% by adding 10 wt % of hollow TiO_2_ NFs, which is 62% higher than that of pure ZnO NPs-based DSSCs.

#### 3.3.2. Counter Electrodes

In the DSSCs, the counter electrode is a transparent conducting oxide coated with a thin layer of Pt, which plays an important role on transferring the electron to the external circuit and regenerating the I3^−^ into I^−^ by reduction in the electrolyte system [193]. However, Pt is an expensive metal, and the corrosive I^−^/I3^−^ redox couple can reduce its catalytic activity [194]. To address these issues, many studies have been carried out on the alternatives to replace the Pt. For example, Qiao et al. [195] fabricated CNFs by electrospinning using an electrocatalyst and low-cost alternative to Pt for I_3_^−^ reduction in DSSCs. The short circuit current density and open circuit voltage of CNF-based cells were comparable to Pt-based cells, while the conversion efficiency was slightly lower than that of Pt-based cells. The hollow activated CNFs with a mesoporous structure were reported, which can promote the electron and ion transfer, decrease the resistance of charge transfer and increase the contact area between the liquid electrolyte and the electrode [196,197]. The resulting overall conversion efficiency of hollow activated CNF-based cells is comparable to that of Pt-based cells. To enhance the electrocatalytic activity, some of the teams prepared transition metals/CNFs [198,199,200,201,202] and transition metal carbides/CNFs [203,204]. The overview of electrospun NFs as a counter electrode material of DSSCs is summarized in Table 4.

#### 3.3.3. Electrolytes of DSSCs

**Figure 8:**
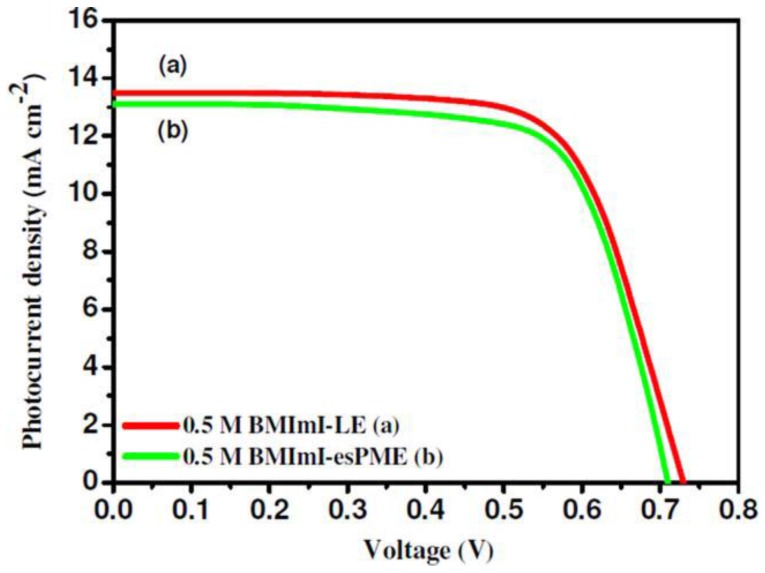
Photocurrent density-voltage (J-V) curves for the DSSC containing (**a**) 0.5 M 1-butyl-3-methylimidazolium iodide (BMImI)-LE and (**b**) 0.5 M BMImI-esPME. Reproduced with permission from [209]. Copyright Wiley, 2015.

The electrolyte is an indispensable component in the DSSCs, which plays an important role in reduction of the oxidized dye by a suitable redox couple and the assistance of the charge transfer from the counter electrode to the photoanode [205]. In the DSSCs, the traditional organic liquid electrolytes and ionic liquid electrolytes exhibit a poor long-term stability because of their being prone to leakage and volatility [206]. Therefore, polymer electrolytes have been developed due to their high ionic conductivity, high thermal stability and good permeability [207]. Kim et al. [208] prepared the PVdF-HFP via the electrospinning method, which showed high electrolyte uptake and ionic conductivity of 653 ± 50% and 4.53 ± 1.3 × 10^−3^ S/cm, respectively. Electrospun PVdF-HEP NFs with different diameters were further discussed [206]. The PVDF-HFP NFs prepared from a 15 wt % spinning solution showed high ionic conductivity (1.295 S/cm) and electrolyte uptake (947%). Dyson et al. [209] soaked electrospun PVdF-HFP membrane (es-PM) in the ionic liquid electrolyte containing 0.5 M 1-butyl-3-methylimidazolium iodide (BMImI), 0.5 M LiI, 0.05 M I_2_ and 0.5 M 4-tert-butylpyribine to obtain the electrospun PVdF-HFP membrane electrolyte (esPME). The 0.5 M BMImI-esPME exhibited better performance than 0.5 M BMImI containing liquid electrolyte (LE), as shown in Figure 8. The poly(vinylidenefluoride-cohexafluoropropylene)/polystyrene (PVdF-HFP/PS) [210] and PVdF-HFP/cobalt sulfide (CoS) [211] were also prepared by the electrospinning method, which showed the good photovoltaic performance in DSSCs. SiO_2_ NF/PEO-(PVdF-HFP) was also synthesized via electrospinning, which possesses high ionic conductivity of 9.9 × 10^−4^ S/cm [212]. In addition, SiO_2_ NF/PEO-(PVdF-HFP) as the polymer electrolyte can enhance the stability of the interface between the electrolyte and the semiconductor electrodes. The photovoltaic performances of electrospun NFs electrolytes are summarized in Table 5.

### 3.4. Supercapacitors

As a promising novel energy storage device, electrochemical capacitors (supercapacitors) have been used in various fields because of their high power density, fast charging and discharging times and long cycle life [215]. According to different energy storage mechanisms, supercapacitors are classified into pseudocapacitors and electric double-layer capacitors (EDLCs) [216]. The pseudocapacitors involve a faradic charge storage process that was facilitated by a redox reaction at the interface between electrode and electrolyte [217]. The EDLCs store electrical energy via the accumulation of electric charges at the electrical double layer formed at the electrode-electrolyte interface [218].

In the EDLCs, the capacitance depends on the surface area accessible to the electrolyte, and porous carbon has been investigated because of its high surface area [219]. Electrospinning techniques are one of the most facile methods to fabricate high PCNFs [27,220,221,222,223]. Yan et al. [224] prepared a light-weight CNF/graphene nanosheet (CNF/GNS) composite paper by electrospinning. The CNF/GNS composite paper showed a high specific capacitance of 197 F/g, about 24% higher than that of pure CNF paper. CNT/CNFs [225] and graphene-beaded CNFs (G/CNFs) [226] were synthesized via electrospinning techniques. The performance of pure CNFs is significantly improved after adding CNT and graphene. Song et al. [227] fabricated nitrogen-enriched PCNF by electrospinning. The nitrogen-enriched PCNF possessed a high specific surface area, good thermal stability and high electrical conductivity, which exhibited the maximum specific capacitance of 251.2 F/g. The transition metal oxide encapsulated on electrospun CNFs, including Fe_3_O_4_/CNFs [228], Co_3_O_4_/CNFs [229], Fe/CeO_2_/CNFs [230], MnO_2_/CNFs [231,232], MnO*_x_*/CNFs [233], NiO/RuO_2_/CNFs [234], RuO_2_/ACNFs [235], ZnO/ACNFs [236] and V_2_O_5_/CNFs [237], was synthesized via electrospinning, and all exhibited good electrochemical performance.

Besides, transition metal oxides are also promising pseudocapacitors electrode materials, due to their high electrical conductivity, several possible oxidation states and electrochemical stability. Kim et al. [238] fabricated electrospun RuO_2_ NF mats to support the RuO_2_ NPs. RuO_2_ NF mats with RuO_2_ NPs displayed high specific capacitance of 886.9 F/g at a scan rate of 10 mV/s and a low capacity loss of only 30% from 10 to 2000 mV/s. Electrospun V_2_O_5_ NFs [134] and Mn_3_O_4_ NFs [239] were also demonstrated to be promising electrode materials for pseudocapacitors. Wang et al. [240] fabricated the NiO NFs modified by citric acid (NiO/CA) with a hollow tube via electrospinning techniques. The pseudocapacitors with NiO/CA as electrodes displayed superior capacitive performance with large capacitance (336 F/g) and excellent cyclic performance (the capacitance decreases only by 13% of the initial capacitance after 1000 cycles). α-Fe_2_O_3_ with porous fiber and α-Fe_2_O_3_ with nanograin structures were prepared by the electrospinning technique [241], which were used as electrode materials in supercapacitors. The supercapacitors with both showed high capacitance retentions even after 3000 cycles. Electrospun 1D porous bimetallic ZnCo_2_O_4_ NTs showed a much higher specific capacitance of 770 F/g at 10 A/g and good cycling stability of only a 10.5% loss after 3000 cycles [242]. Lin et al. [243,244,245] reported La*_x_*Sr_1 − *x*_Cu_0.1_Mn_0.9_O_3 − δ_ (0.1 ≤ *x* ≤ 1) NFs, La*_x_*Sr_1 − *x*_CoO_3 − δ_ (0.3 ≤ *x* ≤ 1) NFs and La*_x_*Sr_1 − *x*_NiO_3 − δ_ (0.3 ≤ *x* ≤ 1) NFs, which exhibited excellent electrochemical performance. The supercapacitors with La*_x_*Sr_1 − *x*_CoO_3 − δ_ (0.3 ≤ *x* ≤ 1) NFs as the electrode exhibit the highest specific capacity of 747.75 F/g at a current density of 2 A/g.

## 4. Conclusions

In this review, we summarized some progress in electrospun NFs for energy applications, such as lithium-based batteries (LIBs, LSBs and Li-O_2_ batteries), fuel cells, dye-sensitized solar cells and supercapacitors. As the materials for electrodes and separators in lithium-based batteries, electrospun NFs exhibited high power capabilities and good kinetic properties because of the decreasing length of Li^+^ diffusion pathways, which is mainly attributed to their high specific surface areas and high porosities. In fuel cells, electrospun NFs were used as the electrode materials and electrolyte membranes. Being electrocatalysts for electrode materials, electrospun NFs showed high activities and good durabilities. As electrolyte membranes, electrospun NFs exhibited high electrolyte uptakes and ionic conductivities. Equally, in terms of the application in dye-sensitized solar cells, the photoelectrodes prepared by electrospun NFs demonstrated high photoelectric conversion efficiencies due to their unique fiber morphologies. Supercapacitors with NFs exhibited high specific capacities and good electrochemical performances, which result from the high specific surface areas, good thermal stabilities and high electrical conductivities of NFs. As a promising synthesis technology for NFs with various morphologies, electrospinning has been largely explored. Nonetheless, the improvement of the accuracy of the experimental parameters and the development of NFs with multiple structures are currently being undertaken.

## Figures and Tables

**Table 1:** Electrospun cathode materials for Li-ion batteries (LIBs) and the corresponding electrochemical performance. DC: Discharge capacity.

Material	Diameter/μm	Structure	DC (after Cycle Number)/mAh·g^−1^	Rate	Reference
LiCoO_2_	0.5–2	fiber	123 (20)	20 mA/g	[41]
LiCoO_2_–MgO	1–2	core-shell fiber	163 (40)	20 mA/g	[42]
LiNi_0.5_Co_0.2_Mn_0.3_O_2_	0.3	particle	160 (5)	20 mA/g	[45]
LiNi_1/3_Co_1/3_Mn_1/3_O_2_	0.1–0.8	nanofiber	116 (30)	85 mA/g	[46]
Li_1.2_Mn_0.54_Ni_0.13_Co_0.13_O_2_/CNFs	－	nanofiber	176.7 (100)	1 C	[47]
Li_1.2_Ni_0.17_Co_0.17_Mn_0.5_O_2_	0.1–0.5	nanofiber	141–205 (20)	14.2 mA/g	[48]
LiFePO_4_/C	－	fiber	131–145 (0.1)	－	[51]
LiFePO4/CNT/C	0.168	nanofiber	169 (average)	0.05 C	[52]
LiFePO4/CNT/C	－	core-shell fiber	－	－	[53]
V_2_O_5_	0.2–0.5	nanofiber	240 (25)	0.1 mA/cm^2^	[56]
V_2_O_5_	0.2–0.4	hierarchical porous nanofiber	133.9 (100)	800 mA/g	[57]
V_2_O_5_	0.1–0.2	hierarchical nanowire	201 (30)	30 mA/g	[58]
V_2_O_5_	0.3–0.5	porous nanotube	130.5 (50)	0.2 C	[59]
LiMn_2_O_4_	0.17	porous network	146 (1)	0.1 C	[60]
Li_3_V_2_(PO_4_)_3_/CNF	0.3	nanofiber	102 (1000)	20 C	[61]
Li_3_V_2_(PO_4_)_3_/CNF	0.18–0.43	nanofiber	118.9 (1000)	20 C	[62]
LiNi_0.5_Mn_1.5_O_4_	<0.2	nanofiber	138 (1)	50 mA/g	[63]
LiNi_0.5_Mn_1.5_O_4_	0.05–0.25	nanofiber	120 (50)	0.01 mA/cm	[64]
LiNi_0.5_Mn_1.5_O_4_	0.05–0.1	nanofiber	300 (1)	27 mA/g	[65]

**Table 2:** Electrospun anode materials for LIBs and the corresponding electrochemical performance.

Material	Diameter/μm	Structure	DC (after Cycle Number)/mAh·g^−1^	Rate	Reference
Carbon	0.2–0.3	nanofiber	350	100 mA/g	[66]
Carbon	0.3–0.4	nanofiber	255 (200)	0.2 A/g	[67]
Carbon	0.1–0.2	porous nanofiber	1780 (40)	50 mA/g	[68]
Carbon	0.9–1.2	hollow nanofiber	900.6 (1)	100 mA/g	[69]
Ag/C	~0.3	hollow nanofiber	600.15 (100)	50 mA/g	[70]
GeO*_x_*@C	1	core-shell	875 (400)	160 mA/g	[71]
Co_3_O_4_/CNF	0.8–1.2	core-shell nanowire	795 (50)	100 mA/g	[73]
Mn_3_O_4_/CNF	0.5–1	hierarchically mesoporous	760 (50)	100 mA/g	[74]
N/GeO_2_-CNFs	0.075	nanofiber	1031 (200)	100 mA/g	[75]
Si/C	2–5	hollow fiber	725 (40)	0.2 A/g	[78]
Si/C	0.6–0.8	porous nanofiber	870 (100)	0.1 A/g	[79]
Si/C	—	hollow fiber	1045 (20)	100 mA/g	[80]
Si/C	0.186	nanofiber	1215.2 (50)	600 mA/g	[81]
Si/C	~2	core-shell	603 (300)	0.5 A/g	[82]
Si/C	~0.2	Fiber paper	1267 (100)	500 mA/g	[83]
Sn/C	0.1–0.18	core-shell	546.7 (100)	40 mA/g	[85]
Sn/C	~0.2	porous nanofiber	774 (200)	0.8 A/g	[87]
Sn/C	0.15–0.25	Hollow nanofiber	737 (200)	0.5 C	[88]
TiO_2_	~0.119	nanofiber	176.7 (100)	0.1 C	[91]
TiO_2_	~0.5	fiber-in-tube	177 (1000)	200 mA/g	[92]
Co_3_O_4_/TiO_2_	~0.3	hierarchical	602.7 (480)	200 mA/g	[93]

**Table 3:** Electrospun separators for LIBs and the corresponding electrochemical performance. PVdF: poly(vinylidene fluoride); HFP: hexafluoropropylene; TEGDA-BA: triethylene glycol diacetate-2-propenoic acid butyl ester; PEO: polyethylene oxide; LLTO: lithium lanthanum titanate oxide.

Material	Diameter/μm	Conductivity/mS·cm^−^^1^ (T/°C)	DC (after-1st)/mAh·g^−^^1^ (Rate/C)	Anodic Stability Voltage/V (vs. Li/Li^+^)	Reference
PVdF	1–1.65	1.0 (25)	－	4.5	[103]
PVdF-HFP	1	1.0 (20)	136–142 (0.1)	4.5	[104]
PVdF-HFP	0.512–0.710	6.16 (20)	138–184 (0.2)	5.0	[105]
PVdF-HFP/SiO_2_	2–5	4.3 (25)	139–170 (0.1)	－	[106]
PVdF-HEP/SiO_2_	1–2	8.06 (20)	153–170 (0.1)	>4.5	[107]
PVdF-HEP/PMMA	0.200–0.350	2.0 (25)	133.5–145 (0.1)	－	[108]
PVdF-HEP/PAN	0.320–0.490	1.0 (25)	131–145 (0.1)	>4.6	[109]
PVdF/SiO_2_-PAALi	0.750	3.5 (25)	151–156.5 (0.1)	5.05	[110]
PAN	0.350	2.14 (25)	135–150 (0.1)	>4.7	[111]
PAN	0.880–1.260	1.7 (20)	108–113 (0.5)	－	[112]
PAN-PMMA	0.2–1.0	5.1 (－)	127–135 (0.1)	5.2	[113]
PAN/SiO_2_	0.8–1.4	2.8–3.6 (－)	−163(0.2)	4.75	[114]
PAN/SiO_2_	0.100–0.300	11 (－)	127–139 (0.5)	5.0	[115]
PAN/Al_2_O_3_-TEGDA-BA	3–5	2.35 (25)	240.4 (0.1)	>4.5	[116]
PAN-TEGDA-BA	0.182	5.9 (25)	127–163.6 (0.1)	>5.0	[117]
PAN-PEO	0.250–0.330	5.36 (－)	134 (0.1)	－	[118]
PAN-LLTO	0.250	1.95 (25)	148–162 (0.2)	5.0	[121]

**Table 4:** Photovoltaic performance parameters of dye-sensitized solar cells (DSSCs) with electrospun fibers. FF: fill factor = *V*_max_ × *J*_max_/*V*_oc_ × *J*_sc_.

Material	Diameter/nm	V_oc_/V	J_sc_/mA·cm^−^^2^	FF (%)	η (%)	Reference
CNF+Pt NPs	250	0.83	12.66–13.33	65.6–68.8	7–8	[193]
CNF	250	0.76	12.6	57	5.5	[195]
H-ACNF	190/270	0.73	14.5	62	6.58	[196]
Meso-HACNF	200/360	0.73/0.74	15.4/15.3	64/61	7.21/6.91	[197]
CNF+Co Cr NPs	－	0.685	8.784	54	3.27	[198]
CNF+Co Ni NPs	－	0.74	11.12	54	4.47	[199]
CNF+Co Pd NPs	150	9.8	0.705	36	2.5	[200]
CNF+Cu Ni NPs	－	0.70	7.67	65	3.5	[201]
CNF+Fe Ni NPs	230	0.72	10.1	65	4.7	[202]
CNF/TiC	280–300	0.73/0.72/0.72	9.29/9.71/9.56	62/64/63	4.2/4.5/4.3	[204]

**Table 5:** Photovoltaic performance of the DSSCs based on electrospun fibers.

Material	V_oc_/V	J_sc_/mA·cm^−^^2^	FF (%)	η (%)	Reference
PVdF-HFP	0.75	12.3	57	5.21	[208]
PVdF-HFP	0.78/0.76/0.77	5.759/6.028/5.378	66.18/68.05/66.27	2.98//3.13/2.75	[206]
BMImI-esPME	0.71	13.10	69	6.42	[209]
PVdF-HFP/PS	0.76	11.8	66	5.75	[210]
PVdF-HFP/CoS	0.73	14.42	70	7.34	[211]
PEO-PVdF-HFP-SiO_2_	0.58/0.60/0.59	13.37/13.63/13.18	60.24/59.54/59.67	4.68/4.85/4.66	[212]
PVdF-PAN-Fe_2_O_3_	0.77	10.4	62	4.9	[213]
PVdF-PAN-V_2_O_5_	0.78	13.8	72	7.75	[214]
